# Learning operational strategies in surgery training

**Published:** 2014-04

**Authors:** SHAHRAM PAYDAR, ZAHRA GHAHRAMANI, SHAHRAM BOLANDPARVAZ, HOSSEINALI KHALILI, HAMID REZA ABBASI

**Affiliations:** 1 Trauma Research Center, Shahid Rajaee (Emtiaz) Trauma Hospital, Shiraz University of Medical Sciences, Shiraz, Iran;; 2 Department of Neurosurgery, Shiraz University of Medical Sciences, Shiraz, Iran

**Keywords:** Learning, Strategy, Surgery, Training

## Abstract

**Introduction**: Education and training in surgery is in the middle of apprenticeship style of learning especially in operating room with little importance of understanding on how trainees learn.

**Methods**: This training is one of the most difficult types of training. Medical training and expertise are the specialty of this education system. We can name these complex fields as “Operational Strategies”. The strategies are includes of “what to do”, “what to think” and “what to create”. These strategies are good to test and train higher functions in persons who have professional’s positions.

**Results**: Most of educational fields are complex. It means that the training is not limited in an area and includes of theory fields, areas of decision making and areas of handy and practical skills. These fields are the most relevant skills or expertise which individual must be informed of the performance of maintenance and repair or upgrade and make a new system.

**Conclusion:**  The operational strategy is a new training strategy for surgery students. It is useful to train surgery students to modify and improve their practices and doing surgeries and treating patients in best conditions.

## Introduction

A good training style may not produce the best outcome. Surgical training covers a range of topics, practical knowledge of patients and disease which continue with very important diagnostic and therapeutic basics and to select appropriate procedures. These procedures are appropriate to the circumstances. Normally, others think that surgical training only includes training procedures (1).

The purpose of training style in surgery is to train broad-based and highly qualified surgical assistants to provide the best care of patients with a wide range of diseases and surgical operations strategies.  There are some general surgical programs designed worldwide to ensure that surgical assistants achieve qualification in knowledge and skill both operatively and non-operatively in different surgical conditions (1).

Today, surgeons need to get knowledge and skills beyond the classic teaching models to cover the variety of topics such as leadership, health care management and so on (1).  Also, surgery students and residents need to develop a number of strategies and skills which cannot be achieved unless mastering these strategies; it is good to provide both students and tutors with learning approaches and strategies to enhance deep learning (2).

Although surgical training has changed little since its formation of Halsted at the beginning of the last century, training programs continue to gain high standards because they are monitored, evaluated and structured. It is recommended to teach surgical training to the surgical assistants in the context of patient care. For this reason, we need a major redesign of surgical training and allocation of sufficient resources to achieve the best outcome. Giving this educational system is essential for producing the next generation of highly educated surgeons, increasing the quality of advanced patient care in clinics and teaching hospitals is essential. Also, we need to develop appropriate strategies to implement the recommendations (3).

Successful surgery requires study, clear thinking, technical skills and advanced planning. Wrong operation at the wrong time could be disastrous. To achieve good results of a surgery, a surgeon should develop a concept which includes analysis of idea, study and understanding of the hypothesis of operation (4).

In educational fields, students should be familiar with the structure and function of the system, understand the pathology system disorders, know the different ways to diagnose problems in terms of management systems and proof of pain, and need to be aware of how to repair system problems. This may require learning some skills. Therefore, these strategies can be taught at three levels. 

*Surgery Training Points*

- Surgery training will increase students’ self-efficacy
- The training in surgery is more beneficial for students with low self-efficacy score
- Further studies are needed to verify the change in self-efficacy on patient safety and satisfaction
- Using appropriate operational strategies in surgery has the potential to raise learning opportunities for surgery residents and their training
- These operational strategies are good for surgery residents and will help them to improve skills and knowledge in their field

## Methods

In this study, the advantages and consideration of implementing learning in surgery training are discussed with some of the surgery tutors, teachers and specialists and the following strategies are extracted from these discussions and debates. We feel that these are effective strategies for surgery training.

***Classification of Operational Strategies***

- *What to do*
  First, we should teach the trainee “what we should know to do our job well”. It means how it should be done or what to know to be able to do the job without changing skills. At this level of education, variation is not considered and normally the most common modes are considered. Individuals trained do not usually handle problems outside the learning environment. Therefore, the trainees will be taught by desired skills in less time and the most direct possible way of training usually without addressing the causes and mechanism or other possible modes.
- *What to think*
  The second level of education is “What to think” and we should teach the trainee the points to enable him/her to make the best choice among the available options. At this level of education, the trainee will be taught by specific skills, terms of skills and different ways of diagnosis. Also, they learn skills recognition; choose the best solution and learn how to do it. Therefore, the trainee will be taught different situations and skills in every position. Normally, learning “how it works” by different guidelines will be set at this level.
- *What to Create*
  The third level of education is “what to create”. We should teach the trainee who knows the concepts how he/she can increase his/her knowledge and skills. At this level, creativity is an expression and trainee will be educated and learn to understand the different conditions, implementation of the right skills and also ability to develop new skills they did not learn before. Type of education includes explaining the mechanisms and principles as well as a description of the problems, principles and rules for the system described. In addition to the cases described, they will learn understanding the conditions even in situations which were not taught by tutor.

## Results

At the first level of education, training directly involves the principles and requirements of each procedure. This level of education will occur in classrooms but it can happen anytime when there is a direct relationship between the learner and the educator.  In this pervasive mode, the learner could hear and see a specific condition or treatment of disease. This is the fastest way of education but deep learning and skill levels are often very low. 

At the second level of education, the trainee will learn different conditions and types of strategies which we discussed at the first level and apply them in the appropriate conditions. In fact, he/she is taught a different condition and the ability to select the best choice. Indeed, training in the application is the thinking education in better conditions of features and limitations. In learning theories, there are various diagnostic and treatment algorithms. Also, surgical assistants will experience different conditions with experienced tutors and more experienced people and internalize various conditions, environmental constraints and the different decisions people take. 

The most important thing to teach the surgical assistant is “Motivation is always better”.  At the third level, training should be based on learning style and getting the information sources, principal conclusions and applying what they learned and created in different conditions. Therefore, they will get the ability of learning, creating and promoting knowledge with environmental features and constraints without direct presence of an instructor. 

## Discussion

Learning is defined as a permanent change in attitude and behavior as a result of experience. It enables persons to change circumstances and is crucial in health care; whether for patients and their families to adapt to medical conditions and for students and residents to obtain the skills and information necessary to become good surgeons. Learning is more effective to educate surgeons and treating patients (5).

Traditional education has focused on “teaching the correct thing”. It is not a model for teaching that allows working through errors. In making errors, the student can learn to recognize when errors occur and avoid doing it or correct the error before developing it (6).

Some of the learning styles and strategies promise surgeons and practitioners a simple solution to the complex problems of improving the motivation and attitude of the students. Tutors need to try new techniques and strategies to be able to help residents to meet their targets better. To growing interest in learning style and strategies, tutors should begin to explore the highly complex nature of teaching and learning. 

The operational strategy in surgery is a new training strategy for surgery students and residents. 

All the sections of these strategies are in correlation with one another and in sequence. It means that students and residents in surgery should learn “what to do” and “what to think” first and then learn “what to create”. The training goal is to achieve a degree of creativity and do a good job at the right time. Students should learn all the time, proactively respond to the training and learn to re-evaluate their own work and the results of the work to reach to level 3 “what to create”.

Different strategies for managing the condition are expressed in terms of the principles and rules. This enables the learner to develop an operational strategy and planning and implement the best possible job in circumstances even in situations not previously experienced in theoretical or practical surgery.  

In these strategies we can teach the most stable and abstract concepts in the most practical ways. Also, we can teach the trainee everything including disease type, diagnostic and treatment methods in which he/she is a spectator, supervisor or partner. Education will be more effective and will increase the depth training and level of skills. Therefore, they will develop their creativity and ability to flourish undetected problems.

## Conclusion

We recommend the above learning strategies to be used to train surgery students to improve practice and doing surgeries and treating patients in the best possible way. The positive recommendation is that these strategies may prove to be a catalyst for students and residents in surgery department.

**Recommendations**

- Make sure all trainees receive common topics in basic principles of operational strategy concepts, patient care and surgeries.
- New teaching technologies should be introduced as they become available and validated. A goal should be defined the curriculum for surgical skills that should be gained by surgical trainees outside the operating room before they start the surgery.
- It is recommended to have a modular format for training in either general surgery or surgical leading to a relevant.

## References

[1] Collins JP (2008). New standards and criteria for accreditation of hospitals and posts for surgical training. ANZ J Surg.

[2] Azer SA, Guerrero AS, Walsh A (2013). Enhancing learning approaches: Practical tips for students and teachers. Med Teach.

[3] Debas HT, Bass BL, Brennan MF, Flynn TC, Folse JR, Freischlag JA (2005). American Surgical Association Blue Ribbon Committee Report on Surgical Education: 2004. Ann Surg.

[4] Chassin JL (1980). Concept and Strategy in Surgery. Operative strategy in general surgery.

[5] Braungart MM, Braungart RG (2003). Applying learning theories to healthcare practice.

[6] Satava RM (2008). Historical review of surgical simulation- a personal perspective. World J Surg.

